# Analysis of the Ibotenic Acid, Muscimol, and Ergosterol Content of an Amanita Muscaria Hydroalcoholic Extract with an Evaluation of Its Cytotoxic Effect against a Panel of Lung Cell Lines In Vitro

**DOI:** 10.3390/molecules28196824

**Published:** 2023-09-27

**Authors:** Alexander Dushkov, Zuzana Vosáhlová, Alexander Tzintzarov, Květa Kalíková, Tomáš Křížek, Iva Ugrinova

**Affiliations:** 1 Institute of Molecular Biology, Bulgarian Academy of Sciences, 1113 Sofia, Bulgariaalexander_imb@abv.bg (A.T.); 2 Department of Physical and Macromolecular Chemistry, Faculty of Science, Charles University, 128 00 Prague, Czech Republickveta.kalikova@natur.cuni.cz (K.K.); 3 Department of Analytical Chemistry, Faculty of Science, Charles University, 128 00 Prague, Czech Republic; tomas.krizek@natur.cuni.cz

**Keywords:** cancer, fungi, alkaloids, HPLC, mass spectrometry, capillary electrophoresis, cytotoxicity, *Amanita muscaria*

## Abstract

The fungus *Amanita muscaria* is universally recognizable for its iconic appearance; it is also widely regarded as poisonous, inedible, and even deadly. In spite of that, there have been documented cases of use of *A. muscaria*-containing preparations against various diseases, including cancer, to no apparent ill effect. The search for compounds that can be used to treat cancer among various plants and fungi has been intensifying in recent years. In light of this, we describe an HPLC HILIC analytical method for the evaluation of the content of the anticancer compound ergosterol (ERG) and the neuroactive alkaloids ibotenic acid (IBO) and muscimol (MUS) that contribute significantly to the unpleasant physiological syndrome associated with *A. muscaria* consumption. A ‘homemade’ *A. muscaria* tincture made using 80-proof rye vodka as the solvent, an *A. muscaria* extract made with a standardized water–ethanol solution as the solvent, and fractions obtained from the second extract via liquid–liquid extraction with nonpolar solvents were analyzed. The study also presents the results of capillary zone electrophoresis with contactless conductivity detection and UHPLC-MS/MS analyses of the IBO and MUS content of the two native *A. muscaria* extracts and an evaluation of the standardized extract’s cytotoxic effect against a small panel of lung cell cultures in vitro. Our results show that the standardized extract has a significant cytotoxic effect and does not contain the compounds of interest in any significant quantity.

## 1. Introduction

With cancer at the forefront of many areas of clinical and biological research, scientists the world over are looking for novel compounds that exhibit potential for effective therapeutic and supportive applications to fight this deadly condition. While much of the research is focused on examining the action of plant-derived compounds, the world of fungi is also an area of rising interest. One fungus that seems to have not been examined for potential anticancer action as much as others have is *A. muscaria* (L.:Fr.) Hook., also known as fly agaric, fly amanita and mukhomor, among others. It is a fungus with an almost cosmopolitan distribution that can be found in woodland habitats in various boreal regions of the Northern Hemisphere. All known subtypes of *A. muscaria*, as well as the related species *A. pantherina* (DC.) Krombh., are widely considered inedible due to their consumption inducing a characteristic set of symptoms sometimes called ‘pantherina-muscaria syndrome’ [1,2]. The syndrome manifests itself with various central nervous system effects, with symptom onset occurring 30–60 min after consumption of the fruiting bodies; those intoxicated may experience drowsiness, euphoria, and dizziness, with occasional nausea and vomiting also reported [3]. Most cases are resolved within 24 h with little more than supportive care [4,5], with no clinical evidence of hepatic damage [6,7], acute kidney injury [7], or other lasting symptoms [1,8] after the intoxication has passed. A study of the effects of an intraperitoneal injection of an aqueous *A. muscaria* extract on various hepatic, serum, and urinary parameters in mice, such as serum cholinesterase activity, serum glucose levels, liver AST and ALT activities, and liver glucose 6-phosphate dehydrogenase and liver glucose 6-phosphatase activities showed that any deviations in these parameters in the bodies of injected specimens return to levels analogous with those of the control groups after 6 to 12 h [9]. Notably, compared to other fungal species, *A. muscaria* contains a very low amount of amatoxins [10], including less than *Cantharellus cibarius* Fr.—an edible and highly desirable fungus; the fly agaric was also the species with the lowest amatoxin content among twelve other tested species of the genus [11]. Amatoxins are cyclic peptides that inhibit the activity of the enzyme RNA polymerase II both in vitro and in vivo [12,13] and are found in high amounts in the deadly poisonous mushroom *A. phalloides* Vaill. ex. Fr. The low quantities of amatoxins in *A. muscaria* are aligned with the observed lack of lasting liver injury after its ingestion by people [14,15,16].

The principal chemical agents behind the manifestation of the ‘pantherina-muscaria’ syndrome have been independently elucidated by several research teams [16,17,18,19,20] and identified as the isoxazole ring-containing alkaloids ibotenic acid (IBO) and muscimol (MUS). Under standard conditions, IBO (IUPAC name (S)-2-amino-2-(3-hydroxyisoxazol-5-yl)acetic acid) is a colorless crystalline substance with a molecular weight of 158.11 g.mol^−1^, readily soluble in water but less so in ethanol and organic solvents [21,22]. In the human body, IBO acts as a nonselective glutamate receptor agonist due to it effectively being a conformationally restricted analogue of glutamate [23,24] and L-glutamic acid [25,26], two major excitatory neurotransmitters in vertebrate central nervous systems. These qualities make IBO a potent neuronal excitant that acts by altering membrane permeability and resting potential [2]. 

MUS (IUPAC name 5-(Aminomethyl)-1,2-oxazol-3(2H)-one, known also as agarin, pantherine) is a colorless crystalline substance, molecular weight 114.10 g.mol^−1^, readily soluble in water but not in alcohol or organic solvents [21,27]. MUS is a selective agonist of γ-aminobutyric acid (GABA) receptors [28,29,30] due to it being a conformationally restricted derivative of GABA [2]. The binding of MUS to the receptors blocks the neuronal and glial reuptake of GABA, increasing serotonin and acetylcholine levels and lowering norepinephrine [31]. Like GABA, MUS is an inhibitor of the central nervous system (CNS) neurotransmission [32] and has been shown to reduce reaction time in a dose-dependent manner when microinjections of it were administered to the red nucleus of cats [33]. 

Both IBO and MUS have been found in clearly detectable concentrations in various *A. muscaria* varieties [34]. Both compounds have been observed to induce a generalized increase in serotonin in the brains of male albino mice and rats after intraperitoneal injection [35]. The psychoactive threshold doses of IBO and MUS have been determined to be between 30–60 mg for IBO and 6 mg for MUS [4], alternatively as 50–90 mg for IBO and 7.5–10 mg for MUS [27]. The IBO content of a fresh, average-sized fruiting body of *A. muscaria* can be as high as 70 mg [36,37]. Both IBO and MUS have been detected excreted in the urine of mice approx. 1 h after ingestion [2,37], with the amount of excreted IBO being ‘substantial’ relative to the ingested dose [37], suggesting that a large amount of the ingested compound does not reach the brain.

There have been reports of an alcoholic tincture prepared from dried *A. muscaria* caps exhibiting a putative anticancer effect in human patients who use it for self-medication [38]; this same tincture has been previously evaluated for cytotoxicity by the authors and established to have a pronounced cytotoxic effect on lung cancer cell cultures in vitro [39]. The therapeutic dosages recommended by Vladimir Vazharov, a Bulgarian mycotherapist and author of the book ‘Medicinal Fungi of Bulgaria’ [38] do not exceed 0.5 mL per day. Compounds with an anticancer effect that have been determined to be present in *A. muscaria* include derivatives of linear (1→3)-α-D-glucans, which have been shown to increase the amount of macrophages in murine peritoneal exudate by more than 50% when administered to mice [40] and the polysaccharides α-D-galactan (GAL-Am) and β-D-glucan (GLC-Am), which also exhibited a proliferation and clonogenic capacity-reducing effect on certain cancer cell lines [41]. Another possible active compound with an anticancer effect is ergosterol (ERG), a sterol found in fungal cellular membranes that aids in maintaining membrane integrity and has been shown to disrupt tumor growth in mice after oral administration [42]. Ergosterol has also been observed to inhibit cancer growth both in vitro and in vivo by upregulating various tumor suppressors [43], downregulating the β-catenin pathway in colorectal cancer [44], as well as by inhibiting tumor related neoangiogenesis [43]. 

The aim of the present study is to evaluate the IBO and MUS content of an alcoholic tincture prepared by Vladimir Vazharov with rye vodka as a ‘homemade’ method of extraction and purported to have had a beneficial effect on the course of illness in cancer patients [38], as well as that of a tincture prepared using a standardized solution of distilled water and ethanol (1:1, *v*/*v*) and a precise ratio of dried fruiting bodies to solvent, referred to as the ‘standardized extract’. It is the opinion of the authors that establishing the IBO and MUS content of a tincture reportedly used by some patients as an adjuvant therapy [38] is an important step in determining its overall effects in vivo and evaluating the risk of potential unwanted neuroactivity. The study also presents an HPLC method for evaluating the extracts’ ergosterol content, since ERG could be a potential active compound behind the observed in vitro cytotoxicity against lung cancer cell lines [39].

## 2. Results

### 2.1. MTT Cytotoxicity Assay

**Figure 1:**
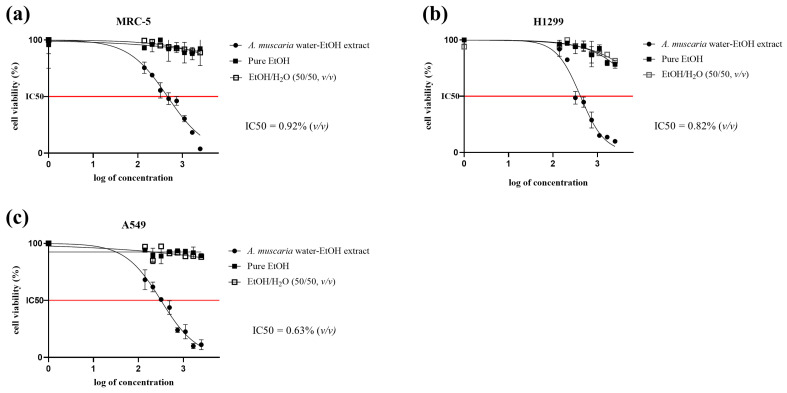
Graphs of the observed decline in cell viability after treatment with increasing concentrations of native *A. muscaria* water–ethanol extract and EtOH or EtOH/H_2_O. (**a**) MRC-5 healthy lung fibroblasts, (**b**) H1299 non-small cell lung carcinoma, (**c**) A549 epithelial lung carcinoma. The volume-to-volume percentage concentrations of extract, pure EtOH, or EtOH/H_2_O added to the cell media are plotted on the X-axes in log format. Each data point represents the mean value of four repeats, and each graph represents three independent experiments.

The MTT cytotoxicity assay (Figure 1) showed that the extract had a pronounced cytotoxic effect on all of the treated cell lines, while the positive controls—both those treated with pure EtOH and those treated with diluted EtOH—did not show any significant decline in viability (with the exception of the H1299 cell line, where a small decline in viability was observed but which was nevertheless not comparable with the effect of the *A. muscaria* extract). 

### 2.2. HPLC Method Development and Optimization

**Figure 2:**
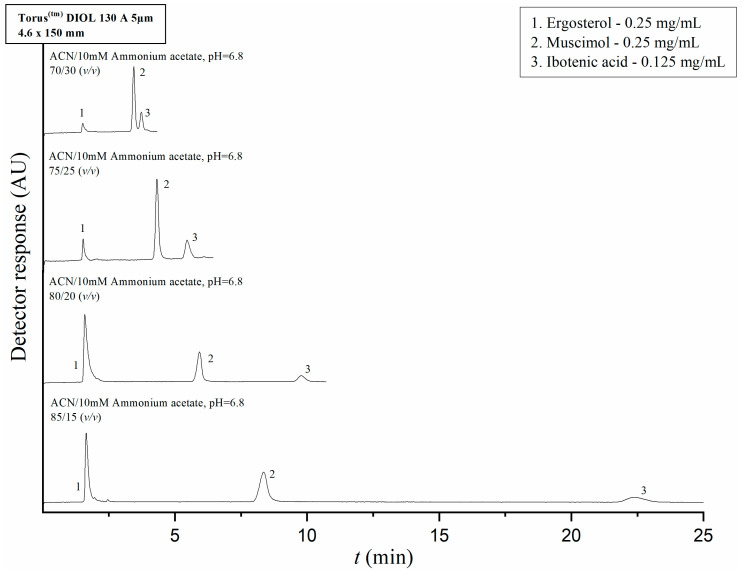
Chromatograms of separation of standards of ergosterol, muscimol, and ibotenic acid evaluated in a Torus^TM^ Diol 130Å 5 μm 4.6 × 150 mm column with a mobile phase consisting of varying isocratic *v*/*v* ratios of acetonitrile and a 10 mM ammonium acetate solution with a pH of 6.8 at a flow rate of 1 mL/min. The injection volume of the mixture was 20 µL; the column temperature was 25 °C and the sample chamber was maintained at 20 °C. UV detection was performed at 255 nm.

The parameters of mobile phase composition and ammonium acetate pH level were optimized with regard to analyte separation prior to analysis of the IBO, MUS, and ERG content of the extracts and fractions. The retention times of the pure compounds in solution were tested on their own, followed by evaluation of the separation from each other of pure IBO, MUS, and ERG in a mixture. We started by evaluating IBO and MUS’ retention and separation using reversed-phase and mixed-mode chromatography; however, these conditions did not provide satisfactory results because the compounds could not be precisely resolved from the dead time marker. Next, IBO and MUS’ retention and separation were tested on a Torus^TM^ Diol 130Å 5 μm 4.6 × 150 mm column (Waters Corporation, Milford, MA, USA) with a mobile phase consisting of pure acetonitrile and a 10 mM ammonium acetate solution with a pH of 6.8 at varying *v*/*v* ratios. The flow rate was set at 1 mL/min. The injection volumes of the analyte solutions and dead time marker solutions were 5 µL and 20 µL, respectively. In the HILIC conditions, IBO, MUS and the dead time marker were sufficiently resolved from each other. The ERG stock solution retention time was also tested on this column; the analyte showed a tendency to elute almost concurrently with the dead time marker, which was not affected by decreasing the polarity of the mobile phase. Finally, a mixture of the three analytes was prepared by mixing 100 µL of the ERG stock solution with 50 µL of the IBO and MUS stock solutions, respectively, for a final concentration of 0.25 mg/mL for ERG and IBO and 0.125 mg/mL for MUS. The chromatograms of separation of standards with varying mobile phase compositions are shown in Figure 2. Upon comparison of the differences in retention times with varying mobile phase composition ratios, a ratio of 80/20 (*v*/*v*) acetonitrile to ammonium acetate was chosen as the mobile phase composition for the analyses of ibotenic acid and muscimol content in the *A. muscaria* extracts and fractions on the basis of optimal peak resolution and overall technical time that would be needed for performing the analysis. Under these conditions, ergosterol eluted at 1.59 min, muscimol at 5.92 min and ibotenic acid at 9.77 min. UV detection was performed at a wavelength of 255 nm.

However, the method was not suitable for analysis of ERG content in the extracts and fractions because ERG elutes almost simultaneously with the dead time marker. For this reason, we tested ERG’s retention on the reversed-phase Atlantis^TM^ dC18 column (5 μm 4.6 × 150 mm, Waters Corporation, Milford, MA, USA) with a mobile phase consisting of pure methanol at a flow rate of 1 mL/min; the analyte was detected at 7.2 min. Performing the analysis for ERG on this column, instead of the diol column, would be optimal.

### 2.3. Application of the Developed HPLC Methods to Amanita Muscaria Extracts and Fractions

**Figure 3:**
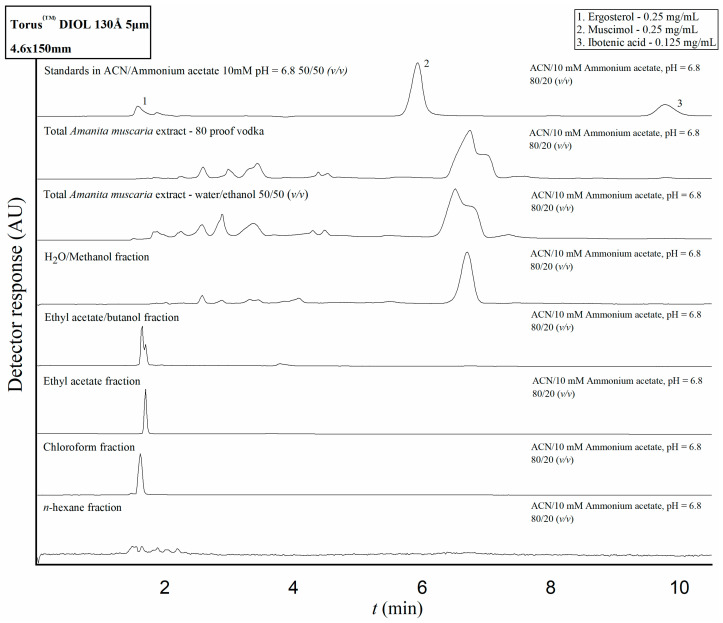
Chromatograms of separation of standards mixture and the total extracts and individual fractions obtained under optimized conditions on a Torus^TM^ DIOL 5 μm 4.6 × 150 mm column. Both extracts and all fractions showed no peaks concurrent with the retention times of IBO and MUS determined using the mixture of standards. UV detection was performed at 220 nm.

Samples of the *A. muscaria* extracts prepared by Mr. Vazharov and the team of IMB-BAS, as well as the fractions obtained from the second extract, were analyzed on the previously described HPLC systems according to the developed methods. Over the course of the analysis no peaks concurrent with the retention times determined for IBO and MUS were detected under the HILIC conditions (Figure 3). Additionally, in all the samples, no peaks were registered around the retention times of IBO or MUS that would have caused possible obscuring of the analytes’ signal by interference issues. Three of the fractions yielded peaks which were concurrent with the determined retention time for ERG; however, their UV absorption spectra were markedly different from the spectrum of ERG. The nonpolar solvents themselves showed a tendency to elute quickly from the diol column, leading to the conclusion that any potential signal related to ergosterol that might have been detected in the fractions would be unresolved with the peaks of the solvents themselves as well as with any other nonpolar compounds that might have been dissolved in them in the process of liquid–liquid extraction.

**Figure 4:**
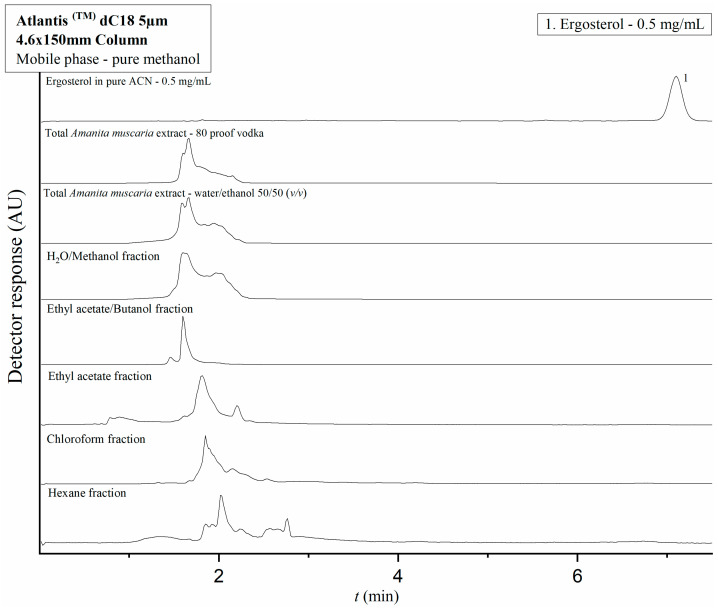
Chromatograms showing the peak of pure ERG and the total extracts and fractions on an Atlantis^TM^ dC18 5 μm 4.6 × 150 mm column with a mobile phase consisting of pure methanol. No peaks concurrent with ERG’s established retention time are observed over the course of analysis for both extracts and all tested fractions.

For that reason, the two extracts’ and the fractions’ ERG content was evaluated separately on the Atlantis dC18 column. (Figure 4) This analysis revealed no prominent peaks concurrent with the determined retention time of ERG under the described experimental conditions. The complete absence of a relevant signal in any of the analyzed extracts and fractions is a likely indicator that the hydroalcoholic extraction method utilized in this study does not yield a product with a concentration of ERG detectable via the method, especially when taking into account the compound’s low solubility in water and ethanol. Based on this finding, it can be assumed that ergosterol is not a major active compound behind the observed in vitro anticancer effect of the *A. muscaria* extracts; it was consequently excluded from consideration during the subsequent CZE/CCD and UHPLC-MS/MS analyses.

### 2.4. Capillary Zone Electrophoresis with Contactless Conductivity Detection Analysis of the Ibotenic Acid and Muscimol Content of the Two A. muscaria Extracts

**Figure 5:**
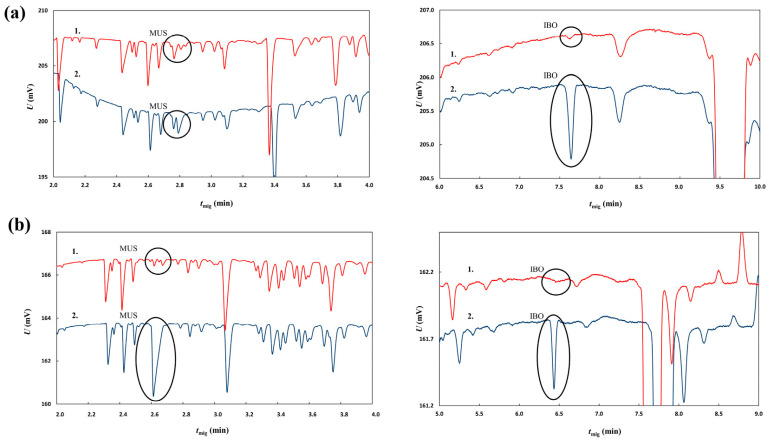
Electropherograms of analyses of the *A. muscaria* extract made with rye vodka (row (**a**); left panel – peaks for MUS, right panel – peaks for IBO) and a 50/50 water–dH2O mixture (row (**b**); left panel – peaks for MUS, right panel – peaks for IBO) as solvent. For each panel, 1. (upper signal) represents the sample of the respective extract diluted with dH2O (1:1, *v*/*v*) and 2. (lower signal) represents the sample of the respective extract diluted with a pure ibotenic acid (0.5 mg/mL) and muscimol (0.35 mg/mL) solution (1:1, *v*/*v*).

As polar compounds that can readily be ionized, IBO and MUS can be separated and determined using capillary electrophoresis. Capillary electrophoresis with capacitively coupled contactless conductivity detection was used as a separation technique based on different separation mechanism to confirm the results of HPLC. A 1M aqueous solution of formic acid (pH = 1.88) was used as the background electrolyte. Under these conditions, the amino groups of both analytes were protonated while the carboxylic group of IBO remained non-dissociated. Both analytes were thus positively charged and they exhibited sufficient migration in the electric field. Both native extracts’ IBO and MUS content was subjected to analysis. Two aliquots were taken from each extract; one of them was diluted with deionized water (1:1, *v*/*v*) and the other was subjected to a 1:1 (*v*/*v*) dilution with a solution of IBO and MUS (concentrations of 0.5 mg/mL and 0.35 mg/mL, respectively) in deionized water. The resulting electropherograms are shown in Figure 5. As the conductivity of the zones of both analytes was lower than the conductivity of 1M formic acid, peaks of both compounds were negative (see Figure 5). The spiked samples showed prominent peaks at around 2.8 min for MUS and 7.6 min for IBO in the vodka extract and 2.6 min for MUS and 6.4 min for IBO for the 50/50 water–ethanol extract. The samples diluted with water without addition of standards exhibited significantly smaller peaks. The migration times for the vodka extract were slightly higher than those observed for the water–ethanol extract. This can be attributed to different matrix effects of both samples. Nevertheless, the migration times of the compounds obtained in measurements of the samples without and with standard addition remained constant for each type of extract. This means that the migration times were repeatable and identification of analytes was reliable. The estimated concentrations of MUS and IBO based on comparison of peak areas from spiked and non-spiked samples were 0.07 mg/mL of MUS and 0.02 mg/mL of IBO for the homemade extract made by Mr. Vazharov with rye vodka as the solvent and 0.02 mg/mL of MUS and <0.01 mg/mL of IBO for the 50/50 (*v*/*v*) water–ethanol extract made at IMB-BAS. The results are in relatively good agreement with those obtained from HPLC-MS/MS. Regarding the fact that no internal standard was used for the MS/MS detection, the results obtained from capillary electrophoresis can be regarded as more reliable thanks to the higher robustness of the conductivity detector signal. The maximum therapeutic dose of the extract is 0.4 mL as per Mr. Vazharov’s book [38]; to ingest the amount of MUS that would reach the psychoactive effect threshold as measured previously [4,27], a patient would have to take between 85.7 and 142 mL of the extract, and to reach the psychoactive threshold of IBO, the volume of the extract would be between 3000 and 4500 mL.

### 2.5. UHPLC-MS/MS Analysis of the Ibotenic Acid and Muscimol Content of the Two A. muscaria Extracts

As HPLC with UV detection could not be used to prove the presence of MUS and IBO in the extracts, a triple-quadrupole tandem mass spectrometer was used as a more sensitive and selective detector. The chromatographic conditions used were identical to the HPLC-UV method. Both positive and negative ion modes were tested for detection of both analytes. The single ion monitoring mode and selected reaction monitoring mode of the triple-quadrupole mass analyzer were tested. For detection of MUS, positive single ion monitoring mode set at *m*/*z* 115.30 was used as it provided the highest sensitivity. Negative selected reaction monitoring mode using the transition of *m*/*z* 157.0 > 113.2 (Q1 pre-bias 18.0 V, Q3 pre-bias 22.0 V and collision energy 11.0 V) was found the most sensitive for the detection of IBO. Quantification of both analytes in the samples was performed using the standard addition method. It should be noted that the estimation of the analyte concentration was based on mere comparison of absolute peak areas of MUS and IBO in non-spiked and spiked samples; no internal standard was used. The results of this quantification must thus be regarded as very rough estimates. The estimated concentrations were 0.02 mg/mL of MUS and 0.04 mg/mL of IBO for the homemade extract made by Mr. Vazharov with rye vodka as the menstruum and 0.005 mg/mL of MUS for the 50/50 (*v*/*v*) water–ethanol extract made at IMB-BAS (Table 1). IBO’s signal was not detected in the latter extract.

## 3. Discussion

All of the analyses performed on the two extracts show that neither using a ‘traditional’ method with vodka as the solvent nor using a water–ethanol tincturing method to perform the extraction yields a product particularly rich in either IBO, MUS, or ERG. IBO and MUS are both known to have been used traditionally and recreationally to induce states of inebriation; however, their presence in a potential *A. muscaria*-derived product used for therapeutic purposes might be considered undesirable or at the very least irrelevant with regards to the putative anticancer effect. With regards to the established threshold psychoactive dosages of both IBO and MUS [4,27], the current findings presented in our study lend credence to the hypothesis that using a tincture made from *A. muscaria* prepared according to the described methods in the therapeutic doses recommended by author V. Vazharov [38] against various cancers cannot induce acute IBO- or MUS-related neurological effects. Unlike acute toxicity, there have not been studies of the possible chronic toxicity that IBO or MUS might induce when taken in low doses over a long period of time; this consideration adds to the study’s significance in establishing the *A. muscaria* extract’s safety and providing a justification for further investigation into the way it enacts its cytotoxic action on cancer cells. While ergosterol (ERG) is a compound known for its notable anticancer properties, it is improbable to be present in an extract prepared using the methods described in this study. Our findings here reveal that the tinctures exhibit a significant cytotoxic effect on lung cancer cell cultures in vitro, indicating the likely presence of unidentified active compounds responsible for these effects. The extract’s low IC50 value against the non-small-cell lung cancer cell line H1299, which is p53-deficient and resistant to multifractionated radiotherapy [45], prompts further investigation into the specific cellular pathways involved and the precise type of cell death that the extract induces. A recent study by Zavadinack and colleagues [41] demonstrated that polysaccharides α-D-galactan (GAL-Am) and β-D-glucan (GLC-Am), extracted from *Amanita muscaria* fruiting bodies, exhibited selective reduction in proliferation against the B16–F10 melanoma cell line. Additionally, another study suggested that commercially sourced *A. muscaria* extract may modulate inflammatory responses in the human microglial cell line HMC3, potentially due to their trehalose content [46]. These findings offer ways to look at the possible molecular pathways involved in the effects in significant depth, if these compounds can be proven to be present in the hydroethanolic extract we have used and isolated for cytotoxicity experiments.

Apart from this, the authors are interested in exploring whether exposure to low doses of the extract (IC50 and below) will affect the rate of cell division and epithelial-to-mesenchymal transition in order to see if the extract would be helpful with controlling metastases and the spread of tumors within the body in vivo. The fractions analyzed in the current study will also be tested for their cytotoxicity in order to narrow down the possible active compounds in the extract; after this, more in-depth analytical techniques such as NMR will be in order, so as to obtain a full picture of the chemical species present and establish whether or not the cytotoxic action is due to single compounds or if there is some kind of synergistic effect that leads to the final outcome. The possibility that the cytotoxic effect of each fraction alone would be less than that of the full extract is also an interesting prospect, as it will suggest a complex, multi-faceted mode of action possibly involving multiple types of cell death receptors and/or disruption of normal processes. The *A. muscaria* extract’s relationship to reactive oxygen species (ROS)—both the possible antioxidant and oxidative effect it may have—must also be investigated, because cancer cells are known to often contain higher levels of ROS than their healthy counterparts [47]; if the extract has an oxidizing effect, then, at least in part, it would be reasonable to assume that the cytotoxic mechanism would be similar to that of many known chemotherapeutics that count on ROS levels increased above what the cancer cell can tolerate. Such a finding may provide the possibility of an adjuvant ROS-inducing cancer therapy with a possible lower amount of devastating side effects, seeing as the anecdotal reports of self-medications do not include, up to this point, any kind of bad reaction directly connected to the consumption of the extract [38]. If the extract has an antioxidant effect, one of the most important considerations that should be taken is to avoid self-medicating with it in conjunction with ROS-generating chemotherapeutics, as this can possibly compromise the desired results. As a final point of interest, the *A. muscaria* species is known for exhibiting considerable regional variations resulting in the naming of several official subspecies [48]; as of the moment of writing of this paper, the variations described are mostly morphological, and a thorough chemical analysis and comparison between them, both with regards to their alkaloid content and any other possible active compound, including anticancerous ones, has yet to be performed. 

## 4. Materials and Methods

### 4.1. Chemicals

Ammonium acetate crystal hydrate was purchased from Sigma-Aldrich (St. Louis, MO, USA) and acetonitrile (LC-MS grade), methanol (LC-MS grade), deionized water (LC-MS grade), and formic acid were purchased from VWR International (Radnor, PA, USA). IBO, MUS, and ERG were purchased from Sigma-Aldrich (St. Louis, MO, USA) as pure crystalline compounds. Ethanol, n-hexane, chloroform, ethyl acetate, butanol, and methanol were purchased from Chim-spectar Ltd. (Sofia, Bulgaria). MTT (3-(4,5-Dimethylthiazol-2-yl)-2,5-diphenyltetrazolium bromide), streptomycin, and penicillin were purchased from Thermo-Fischer Scientific (Waltham, MA, USA).

### 4.2. Cell Lines and Media

The cell lines used in this study were purchased from ATCC (Manassas, WV, USA) (H1299 catalogue number ATCC^®^ CRL-5803TM, A549 catalogue number ATCC^®^ CCL-185TM, MRC-5 catalogue number ATCC^®^ CCL-171TM). All cell media were purchased from ATCC (Eagle’s Minimum Essential Medium (EMEM), catalogue number ATCC^®^ 30-2003TM, RPMI-1640 Medium ATCC ^®^ 30-2001TM, and Dulbecco’s Modified Eagle’s Medium (DMEM), ATCC ^®^ 30-2002TM). 

### 4.3. Cell Line Cultivation

All cell lines were grown in an incubator at a temperature of 37 °C and in the presence of 5% gaseous CO_2_. H1299 cells were grown in RPMI-1640 medium, MRC-5 cells were grown in EMEM medium, and A549 cells were grown in DMEM medium. Fetal bovine serum was added to all media for a final concentration of 10% (*v*/*v*); a mixture of penicillin (final concentration of 100 IU/mL) and streptomycin (100 mg/mL) was also added to all growth media. 

### 4.4. Amanita muscaria Ethanol Extracts

Two water–ethanol extracts from fruiting bodies of *A. muscaria* were used in this study. One of them was prepared by Mr. Vladimir Vazharov from *A. muscaria* specimens collected by him and his colleagues at various locations in Bulgaria and identified as *A. muscaria var. muscaria*. He discarded the stipes and dried the caps in the sun until they became brittle, after which he performed an extraction on the dried caps using 40% (80 proof) rye vodka as the solvent. The ratio of *A. muscaria* material to vodka was approx. 1:10 (*w*/*v*). The caps were left in the solvent at room temperature for 21 days in the dark, after which the marc was filtered through a funnel with a throat filled with cotton and discarded. The filtered tincture was kept bottled in dark glass vials. 

To prepare the second extract, fruiting bodies of *A. muscaria* were collected in the vicinity of Dolny Pasarel village, Bulgaria, by the team of IMB-BAS and identified as *A. muscaria var. muscaria*. The stipes were removed and discarded and the caps were dried in a ventilated dehydrator at a temperature of 73 °C overnight. After drying, the caps were powdered using a mill and then soaked in a mixture of ethanol and distilled water (50/50, *v*/*v*) for 21 days at a ratio of 1:10 (*w*/*v*—25 g. *A. muscaria* cap powder and 235 mL 50% EtOH) away from light. After the period ended, the marc was filtered out using a 45 μm PVDF filter. The tincture was then kept at 4 °C protected from light in airtight vials for the remainder of the experimental procedures. Both extracts were prepared using caps from fruiting bodies at various stages of development. 

### 4.5. Liquid–Liquid Extraction Procedures

A 65 mL portion of the *A. muscaria* extract prepared by the team at IMB-BAS was placed in a round-bottom flask and the ethanol and water in the sample were evaporated using a rotary evaporator (Büchi 011 RE 121 Rotavapor with a 461 water bath, BÜCHI Labortechnik AG. Flawil. Switzerland) at a water bath temperature of 40 °C and rotor speed 120 RPM for 6 h. The total amount of solvent evaporated from the sample was 57 mL; the weight of the yield was 3.566 g. The reduced extract was then redissolved in 70 mL of distilled water and placed in a separatory funnel. In the funnel, n-hexane was added to the redissolved extract (Hex:dH_2_O 2:1, *v*/*v*) and the funnel was vigorously shaken; after the solvents had separated into two distinct layers, the fractions were decanted in separate containers. The water fraction was then returned to the funnel and mixed with chloroform (Chl:dH_2_O 2:1, *v*/*v*), after which the procedure of shaking and decanting was repeated. The water fraction was subsequently extracted with ethyl acetate (EtAc:dH_2_O 1:1, *v*/*v*), then with a 1:1 *v*/*v* mix of ethyl acetate and butanol (EtAc/Bu:dH_2_O 1:1, *v*/*v*) and then with butanol (Bu:dH_2_O 1:1, *v*/*v*), all according to the same procedure; the funnel was finally washed with methanol for a final H_2_O/Met fraction.

### 4.6. Preparation of Standardized Solutions of the Compounds of Interest

Stock solutions of the pure compounds used as standards—IBO, MUS, and ERG—were prepared. IBO and MUS were dissolved in a 50/50 (*v*/*v*) mixture of methanol and a 10 mM ammonium acetate solution in deionized water, pH = 6.8. ERG was dissolved in pure acetonitrile with additional vortexing and sonication of the sample for five minutes. The concentrations of MUS and ERG in solution were 0.5 mg/mL, while that of IBO was 1 mg/mL. 

For HPLC analysis, a mixture of the three compounds was prepared by mixing 100 μL of ERG stock solution with 50 μL of IBO stock solution and 50 μL of MUS stock solution for a final concentration of 0.25 mg/mL ERG, 0.25 mg/mL MUS, and 0.125 mg/mL IBO. 

For UHPLC-MS/MS analysis, a solution of IBO with a concentration of 0.1 mg/mL and a solution of MUS with a concentration of 0.005 mg/mL were used; the compounds were dissolved in a 10 mM ammonium acetate solution with a pH of 6.8.

### 4.7. Samples and Sample Preparation

All *A. muscaria* extracts and fractions were filtered through a 0.45 μm PVDF filter prior to analysis. 

For HPLC experiments, 200 μL aliquots of each extract and fractions were transferred into respective LC vials and subjected to analysis without dilution. 

For CZE/CCD experiments, two aliquots from each of the two total *A. muscaria* extracts were taken; one aliquot from each extract was diluted with deionized water (1:1, *v*/*v*) and the other with a mixture of IBO and MUS (0.5 mg/mL and 0.35 mg/mL respectively) dissolved in deionized water (1:1, *v*/*v*). 

For UHPLC-MS/MS experiments, 200 μL aliquots of each of the two *A. muscaria* total extracts were transferred into respective LC vials and subjected to analysis without dilution.

### 4.8. MTT Cytotoxicity Assay

An MTT cytotoxicity assay [49] was performed in order to establish the standardized water–ethanol extract’s cytotoxicity against a panel of lung cancer cell lines and compare it to the previously established IC50 values for the same [39]. The cells were seeded in 96-well plates (100 μL of medium/well) at a density of 1 × 107 cells per milliliter. After 24 h of incubation at 37 °C and in the presence of 5% gaseous CO_2_, the cells were treated with the standardized *A. muscaria* water–ethanol (50/50, *v*/*v*) native extract for final concentrations of 5%, 3.3%, 2.2%, 1.48%, 0.98%, 0.65%, 0.43% and 0.29% (*v*/*v* extract/cell medium). Additional wells were treated with pure EtOH in such a quantity as to obtain the same concentrations as those that would be present in the wells treated with the native extract (2.5%, 1.66%, 1.11%, 0.74%, 0.49%, 0.32%, 0.21%, 0.14% EtOH/cell medium *v*/*v*) as a positive control. Since the extract itself was made with 50% EtOH and 50% H_2_O, a final portion of wells was treated with a solution of ethanol diluted with dH_2_O (50/50, *v*/*v*) for final EtOH concentrations in the wells identical to those listed for the pure EtOH positive controls. In this way, a slight dilution of the medium was also introduced, so as to observe its possible effect when treating with the same volume of liquid as that in the *A. muscaria*-extract-treated wells. All extracts and EtOH solutions were filtered through a 0.45 μm filter prior to treatment. After incubating the cells for 72 h in the presence of the extract and the ethanol solutions, the medium was removed and replaced with a phenol-free medium with added MTT in a concentration of 0.5 mg/mL. The treated cells were kept in the incubator for 2.5 more hours, after which the medium was removed and replaced with DMSO aliquots (100 μL/well) to dissolve the formazan crystals that had formed. The absorbance was measured on a Varioscan Lux multifunctional microplate reader (Thermo Fisher Scientific, Waltham, MA, USA) at 550 nm. The extract’s IC50 value was established on GraphPad Prism 6 software; the same was also used to present the data in a graph format. The concentrations of the extract and the EtOH solutions are presented in log format.

### 4.9. Instrumentation and Experimental Procedures

HPLC—The separation procedures for IBO, MUS, and ERG, as well as the analyses of the total extracts and fractions, were optimized and performed on a Waters Alliance 2695 HPLC Separations Module with a Waters 2996 PDA detector running on Empower software (Waters Corporation, Milford, MA, USA). For analysis of IBO and MUS content in the extract and fractions, the final separation conditions consisted of a Torus^TM^ Diol 130Å 5 μm 4.6 × 150 mm HILIC column (Waters Corporation, Milford, MA, USA; the elution was performed under isocratic conditions with a mobile phase composed of acetonitrile and a 10 mM ammonium acetate solution with a pH of 6.8 at a volume ratio of 80/20 (*v*/*v*). The flow rate was maintained at 1 mL/min. Injection volume was 20 µL for the mixture of standards and the total extracts and 5 µL for the fractions. The column was equilibrated with the aforementioned mobile phase composition for 40 min before the first injection. For analysis of ERG content in the total extracts and fractions, the final separation conditions consisted of an Atlantis^TM^ dC18 5 μm 4.6 × 150 mm column (Waters Corporation, Milford, MA, USA) with a mobile phase consisting of pure methanol at a flow rate of 1 mL/min. Injection volume was 5.0 µL for the ERG standard solution and 20 µL for the analyzed extract and fractions. The temperature of the samples was maintained at 20 °C and the column was thermostatted at 25 °C for all analyses. 

CZE/CCD—Electrophoretic experiments were carried out on an Agilent 7100 CE system (Agilent Technologies, Waldbronn, Germany) equipped with a capacitively coupled contactless conductivity detector. An unmodified fused-silica capillary of 20 µm ID and 375 µm OD (Polymicro Technologies, Phoenix, AZ, USA) was cut to 50.0 cm total length (effective length of 35.0 cm). Before the first measurement of the day, the capillary was flushed for 15 min with 1M NaOH and 15 min with water. Between individual runs, the capillary was flushed for 15 min with 1M NaOH, 2 min with water, and 4 min with background electrolyte (BGE) using a pressure of 93 kPa. Background electrolyte consisted of 1M formic acid in water (pH = 1.87) filtered through a 0.45 µm PVDF filter. Samples were injected using a pressure of 5 kPa for 20 s. A voltage of 25 kV (current approx. 7 µA) was applied during the analysis together with a pressure of 10 kPa applied to the inlet vial with the BGE.

UHPLC-MS/MS—For the UHPLC-MS/MS analysis, the Shimadzu UHPLC Nexera X3 coupled with a Triple Quad 8045 tandem mass spectrometer (Shimadzu, Kyoto, Japan) was used. The column, mobile phase, flow rate, and temperature were identical to those employed in the HILIC HPLC analysis. The applied conditions of the electrospray ion source were as follows: nebulizing gas flow: 3 L/min, heating gas flow: 10 L/min, interface temperature: 300 °C, desolvation line temperature: 250 °C, heat block temperature: 400 °C, and drying gas flow: 10 L/min. The MS/MS spectrometer was operated in the positive single ion monitoring mode set at *m*/*z* 115.30 for detection of MUS and in the negative selected reaction monitoring mode using the transition of 157.0 > 113.2 (Q1 pre-bias 18.0 V, Q3 pre-bias 22.0 V and collision energy 11.0 V) for detection of IBO. Quantification of both analytes in the samples was performed using the standard addition method.

## Figures and Tables

**Table 1:** Comparison of estimated IBO and MUS content (mg/mL) in the two native hydroalcoholic *A. muscaria* extracts via CZE/CCD and LC-MS.

Method Used	Concentration in Extract with Rye Vodka—mg/mL		Concentration in Extract with dH_2_O/EtOH (50/50 *v*/*v*)—mg/mL	
	IBO	MUS	IBO	MUS
CZE/CCD	0.02	0.07	ND *	0.02
LC-MS	0.04	0.02	ND *	0.005

* ND—Not Detected.

## Data Availability

All data are available from the authors upon request.

## Sample Availability

Samples of the extracts used in the study are available from the authors upon request.
